# Human fertility after a disaster: a systematic literature review

**DOI:** 10.1098/rspb.2023.0211

**Published:** 2023-05-10

**Authors:** D. Susie Lee, Ewa Batyra, Andres Castro, Joshua Wilde

**Affiliations:** ^1^ Fertility and Well-being, Max-Planck-Institute for Demographic Research, 18057 Rostock, Mecklenburg-Vorpommern, Germany; ^2^ Centre for Demographic Studies (CED), Barcelona, 08193, Spain; ^3^ Institute of Labor Economics (IZA), 53113 Bonn, Germany

**Keywords:** fertility, disaster, humans, systematic literature review, birth counts

## Abstract

Fertility is a key demographic parameter influenced by disaster. With the growing risk of disasters, interest in the fertility response to a disaster is increasing among the public, policy makers and researchers alike. As yet, a synthesis of the current evidence on how fertility changes after disaster does not exist. We reviewed 50 studies retrieved from a systematic search based on a pre-registered protocol. We found an overall negative impact of disasters on fertility. If any, increases in fertility were mostly linked with weather-related physical disasters. We also identified 13 distinct mechanisms which researchers have considered as underlying the fertility effects of disaster. By contrast to the common belief that disasters are more likely to increase fertility in contexts with already high fertility, we found little evidence to suggest that the total fertility rate of the studied populations was an important predictor of the direction, timing or size of fertility impacts. While this may be because no relationship exists, it may also be due to biases we observed in the literature towards studying high-income countries or high-cost disasters. We summarize the methodological limitations identified from the reviewed studies into six practical recommendations for future research. Our findings inform both the theories behind the fertility effects of disasters and the methods for studying them.

## 1. Introduction

Disasters can significantly affect the lives of those who experience them. Beyond the proximate perils of displacement, death, disease and injury, disasters are often accompanied by broader challenges such as economic crisis, reduced access to health facilities and other services, and food insecurity. Since childbearing is one of the most consequential choices people make, it is not surprising that disasters affect whether and when people have children, and how many. With the growing frequency and severity of disasters [1,2] especially due to the global climate crisis [3], how disasters affect fertility has become a topic of interest increasingly shared among the public, policy makers and researchers alike [4,5].

Yet in spite of the importance of and broad interest in the topic, a synthesis of the current evidence on how disasters affect fertility (the number and timing of births) does not yet exist. Doing so is a complex task, for multiple reasons. First, ‘disaster’ is a blanket term for many types of catastrophic events, each of which may vary from one another in their impact on fertility. Second, even within disaster type, the direction and magnitude of the effects thereof differ depending on population characteristics [6]. For example, it was predicted that the COVID-19 pandemic would lead to a reduction in births in high-income, low-fertility contexts, but to an increase in low-income, high-fertility contexts [7]. Third, disasters can have different effects in the short, medium and long runs. Indeed, the very first scientific observation on the topic, made in 1892 [8], noted that fertility dropped around 9–10 months (i.e. the average human gestation length) after the spike of mortality during the 1889–1890 influenza pandemic, and then increased above and eventually converged to the expected average. Although this sequence of initial trough, rebound and stabilization mirrors the temporal dynamics of mortality crises [9], we do not yet know the degree of support for similar fertility dynamics within the existing evidence base. Fourth, the mechanisms underlying fertility change are numerous and complex, making it difficult to differentiate specific pathways by which disasters affect fertility [10]. Lastly, methodological differences hinder the comparison of fertility effects across studies. This is particularly important since methodological quality determines the degree to which an association between disaster and fertility affords causal interpretation, given that experimental study on this topic is nearly impossible.

We conducted a systematic review [11] of studies that quantified the change in live birth counts after disasters, to provide a comprehensive view of the state-of-the-art evidence in this field. Building on existing reviews or continent-level analysis on other catastrophic events such as wars and conflicts, humanitarian crises and economic recession [12–16], we reviewed fertility change after natural or technological disasters. They included disease outbreaks, physical disasters (e.g. earthquake, weather-related events) and technological disasters (e.g. radiation events, poisoning, explosion). We use the term ‘disease outbreak’ to refer to community-level disease occurrence including epidemics and pandemics, similar to the approach taken by the Center for Disease Control (https://www.cdc.gov/outbreaks/index.html). We focused on studies that operationalized disasters as relatively concentrated and unanticipated exposure [17] experienced at the community-level, and thus excluded those that measured exposure at individual-level or with a continuous scale (see Methods for our eligibility criteria).

Based on the literature identified through a predefined protocol, we analysed (1) how disaster–fertility relationships have been studied in terms of spatio-temporal coverage and methodology, and (2) the direction, magnitude and mechanisms by which disaster affects fertility. Given the diversity of methodological approaches and measures employed by the reviewed studies, we deemed the synthesis of effect sizes via meta-analysis infeasible. We instead opted to present a narrative synthesis accompanied by statistical analysis as needed, and offer methodological recommendations for future research.

## 2. Methods

The protocol for this review was pre-registered at PROSPERO (ID: CRD42022320294) before data extraction and analysis. Details on the database search procedure and results, and the data extraction template and data extracted can be found on the project website (https://osf.io/dpgqf/).

### (a) Scope and eligibility criteria

The scope of our review was determined by our two core concepts—disaster [exposure] and fertility [outcome]. Disaster is a difficult concept to define [18] and ranges from everyday emergencies, economic shocks, conflicts and wars, to various types of catastrophic events which may entail distinct patterns of disruptions [19,20]. The present review focused on natural and technological disasters, for which—to the best of our knowledge—no systematic reviews are available. The primary outcome of interest was live birth counts, a measure with a direct impact on population structure and size. We excluded other reproductive health measures beyond live birth counts, given already existing reviews on the sexual and reproductive health consequences of disasters [21] or adverse birth outcomes [22–27].

We selected studies that were: (a) either published or in press in peer-reviewed journals as of early February 2022, (b) on human populations regardless of publication year and languages, as long as (c) information on the direction or magnitude (or both) of the relationship between disaster and fertility was reported. We restricted studies to those on (d) the relatively acute impact of a disaster, not extending to studies that had assessed exposure to post-disaster environments created by a disaster itself, such as displacement camps or food insecurity, or the intergenerational impact of disaster on fertility. We focused on studies which examined (e) one of the disaster types—both natural and technological—listed in the International Disasters Database [28] and (f) fertility in terms of live birth counts, excluding studies that used a combined measure of stillbirth and live birth [29]. Lastly, we reviewed (g) studies that operationalized disaster as a relatively concentrated and/or unanticipated exposure, as explained below.

### (b) Operationalizing exposure to disaster

Different identifications of exposure yield different findings and interpretations of the impact of an exposure [17]. In the present study, we focused on studies that operationalized disaster as a ‘concentrated’—meaning having a major impact on many communities—and/or ‘unanticipated’—meaning seemingly random, non-cyclic and extreme—exposure to entire communities. Doing so would allow interpreting the impact of a disaster as a *population-level fertility response to significantly and unexpectedly abrupt disruptions to routine*. This operationalization of disaster had three implications for the scope of this review. First, exposure to disaster is not reduced to individual-level exposure. For example, we omitted studies that compared fertility between infected and uninfected individuals, as the literature on the fertility effect of HIV commonly does [30,31]. The current review thus approached fertility from a demographic perspective, where fertility is a process by which populations replace themselves, as opposed to a strictly clinical perspective. Second, exposure to disaster should not be intermittent (e.g. seasonal heat waves) or persistent (e.g. endemic diseases). In this regard, identifying a specific disaster onset should be reasonably possible. Third, exposure to disaster is measured entirely at the population-level. We also excluded studies that measured exposure on a continuous scale, such as degree of rainfall [32], seismic intensity scale [33], or the prevalence of a disease [34], which would not allow interpreting the impact of a disaster event as a whole.

### (c) Literature search and study selection

**Figure 1.**
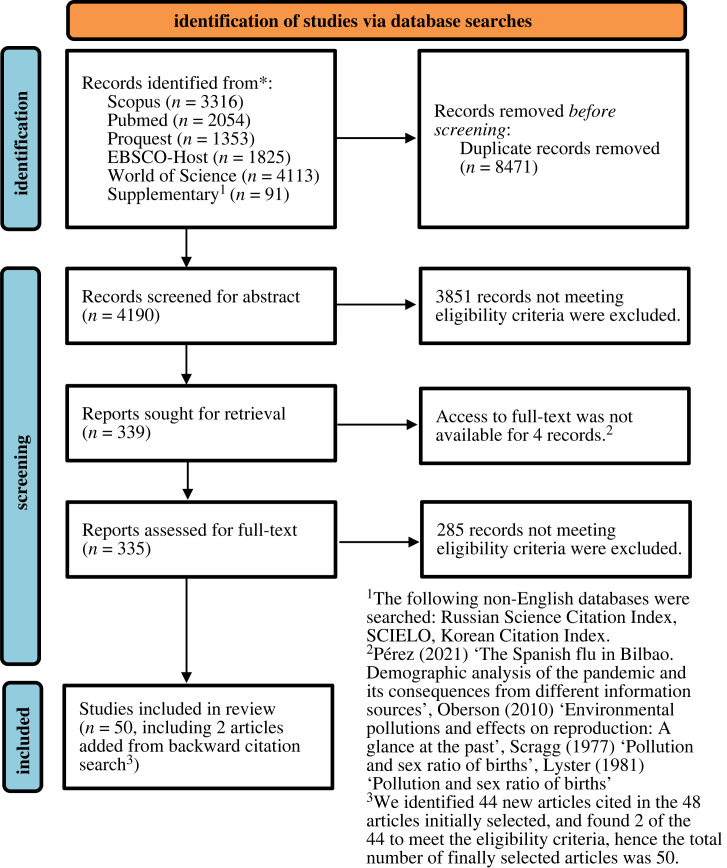
Flow chart of studies selection process.

In February 2022, we searched databases and conducted an additional search in non-English databases for which we had language expertise and access. We used Boolean operators, whenever they were supported by databases, to combine search terms for each disaster and fertility (e.g. ‘hurricane AND fertility’). We searched for the presence of any combinations either in the title or abstract. A total of 12 661 studies were found, of which 4190 deduplicated unique studies were screened (figure 1). We used Covidence, an online record management system for systematic review [35], to conduct screening and data extraction. First, the title and abstract of each article were read by two researchers, who independently rated whether or not a study met the eligibility criteria (yes, no or maybe). Only the studies unanimously voted either ‘yes’ or ‘maybe’ entered the full-text screening. Second, the full text of each article was read by two researchers and rated in reference to the eligibility criteria. In both screening procedures, any discrepancies in assessments were discussed and resolved by the whole team. Third, based on the 48 articles chosen for review, we extracted information on study characteristics, methods and findings. Data were extracted twice each by two researchers, and any discrepancies were resolved in discussion among them. Lastly, during the data extraction, we identified 44 potentially relevant additional articles through backward citation search. Two of these studies met the eligibility criteria and were included for review. We did not perform a forward citation search, since studies on the COVID-19 pandemic are rapidly increasing. We identified 50 studies (figure 1, table 1) which quantified how live birth counts change after disasters.

**Table 1.** Summary of 50 reviewed studies arranged by disaster types, specific disasters within types, and alphabetical order of countries studied. Follow-up lag is marked with an asterisk if it could be approximately estimated due to limited information available from a study. Direction of fertility effects is based on summary effects, which are information from the authors' preferred analysis or mentioned as the main finding of their study. The dash sign (−) means that a study did not provide relevant information.

disaster	country	research design: comparison	sample size	data	follow-up (months since disaster onset)	fertility effects (based on summary effects)	heterogeneity in effects	study (last name of the first author, publication year)
disease outbreaks (48% of the 50 studies)								
measles outbreak	Iceland	within-population	∼70 000–80 000 people	yes	7	↓		Gunnarsdottir [42]
1918 influenza	Canada	within-population	∼10 baptisms per year	no	24*	↓		Herring [59]
1918 influenza	India	within-population	50 000 births	yes	6*	↓		Mills [43]
1918 influenza	Japan	within-population	—	yes	9–10	↓		Chandra—Japan [38]
1918 influenza	New Zealand	within-population	—	yes	12	↓	by race: larger decline in Maori	Wilson [50]
1918 influenza	Taiwan	within-population	—	yes	9–10	↓		Chandra—Taiwan [39]
1918 influenza	US	within-population	—	yes	0–3, 9–10	↓		Chandra [41]
1918 influenza	US	within-population	21 334 births	no	9–10	↓		Dahal [60]
1918 influenza	Denmark, Norway, Sweden, US	within-population	—	unclear	6	↓	by region (urban/rural): Copenhagen versus rural Denmark were similar	Bloom-Feshbach [36]
Measles outbreak	Japan	within-population	—	yes	9–10	↓		Mizumoto [61]
Zika	Brazil	within-population	3 820 304 births	yes	5*	↓	within-region heterogeneities	Castro [62]
Zika	Brazil	within-population	77 977 births	yes	9–10	↓		Diaz-Quijano [63]
Zika	Brazil	within-population	373 136 births	yes	9–10	↓	by regions: larger decline in cities with higher prevalence of microcephaly, but reduction also occurred with no or few cases	Diaz-Quijano [64]
Zika	Brazil	within-population	2 857 800 births	yes	3*	↓	by age, education, regions: larger decline in younger women, highly educated, and in northeast	Marteleto [65]
Zika	Brazil	between-population	all registered births	yes	9–10	↓	by age and education: larger decline in older women and educated	Rangel [66]
Zika	Brazil	between-population	33 366 births	yes	12*	↓	by age: larger decline in younger women	Triaca [45]
Zika	Colombia	between-population	15 680 births	yes	7–10	↓	by age or education: no evidence for heterogeneity	Gamboa [46]
Zika	Singapore	between-population	657 women	no	0	↓	some interactions by age, desired fertility, education, and regions	Tan & Pang [67]
COVID-19	22 high-income countries	within-population	—	yes	9–10	mixed across countries		Aassve [51]
COVID-19	Colombia	within-population	—	yes	−3*	↓	by age: larger decline among 10–14 years	Mendoza [68]
COVID-19	Italy	within-population	1579 (Turin), 2656 (Milan)	unclear	9–10	↓		DeRose [69]
COVID-19	Italy	within-population	4611 births	yes	9–10	↓		Somigliana [70]
COVID-19	Italy	within-population	555 live deliveries	no	6	↓		Trombetta [71]
COVID-19	US	within-population	12 099 births	no	9–10	↓	by regions: larger decline in New York City	McLaren [72]
technological disaster (18% of the 50 studies)								
Kyshtym disaster	USSR	between-population	1063 people	unclear	—	—	by age: lower percentage of people having children among population born at the moment of accident	Buldakov [55]
Iraq methyl mercury poisoning	Iraq	within-population	66 549 births	no	27	↓		Greenwood [73]
Minamata poisoning	Japan	between-population	1 904 534 births	yes	60	↓		Yorifuji [54]
Chernobyl accident	15 former Soviet republics	Within-population	—	unclear	−4	↓		Grech [37]
Chernobyl accident	Belarus	between-population	—	unclear	44	↓	by mode of conception (natural versus artificial reproductive technology)	Shakhot'ko [74]
Chernobyl accident	Italy	Within-population	43 905 births	yes	9–10	↓	by regions	Bertollini [44]
Chernobyl accident	Ukraine	between-population	—	no	8	↓		Donets' [75]
Washington water crisis	US	between-population	—	yes	0	↓		Edwards [52]
Flint water crisis	US	between-population	15 425 births	yes	3	↓	by race: slight reduction in Blacks and increase in Whites	Wang [76]
physical disasters (34% of the 50 studies)								
Avalanche	UK	between-population	445 births	yes	15	↑	by bereavement status: bereaved parents contributing to increased fertility	Williams [77]
Guatemalan earthquake	Guatemala	within-population	—	yes	0	↑	by regions: fertility changes were stronger in the capital	Houdaille [40]
Flood	Poland	between-population	362 280 women	unclear	6*	↑		Neuberg [78]
Tajikistan drought	Tajikistan	Within-population	—	no	7	↓		Clifford [79]
Red river flood	US	between-population	57 007 births	yes	0	↓		Tong [80]
Hurricane Hugo	US	between-population	—	yes	4	↑	by regions: no differential impact by severity of hurricane	Cohan [81]
Hurricane Katrina	US	between-population	all regi stered births	yes	6	mixed across racial groups	by race: decline in Blacks but increase in Whites	Seltzer [82]
Indian earthquake	India	between-population	266 290 women	no	12	↑	by age, education, minority caste group, regions: fertility increases larger in rural areas, among scheduled tribe women, 20–30 years, and uneducated women; fertility reduction in highly educated women	Nandi [83]
Indonesian tsunami	Indonesia	within-population	252 women	no	1	↓		Kinoshita [84]
Indonesian tsunami	Indonesia	between-population	6363 women	no	24	↑	by age: fertility increases larger among 20–24 years	Nobles [85]
Chilean earthquake	Chile	within-population	3279 deliveries	no	1	↓		Oyarzo [86]
Chilean earthquake	Chile	between-population	all registered births	yes	9–10*	↑		Scapini [87]
Japanese earthquake	Japan	between-population	32 078 births	yes	9–10	↓		Hamamatsu [47]
Japanese earthquake	Japan	within-population	∼28 000 births	yes	7*	↑	by regions: fertility decline is larger in regions exposed more to radiation	Körblein [48]
Japanese earthquake	Japan	within-population	—	yes	0	↑		Kurita [49]
Iranian earthquake	Iran	within-population	44 265 women	no	5*	↓		Bahmanjanbeh [88]
Philippine typhoon	Philippines	within-population	—	yes	—	—		Mangada [53]

### (d) Data and analyses

**Figure 2.**
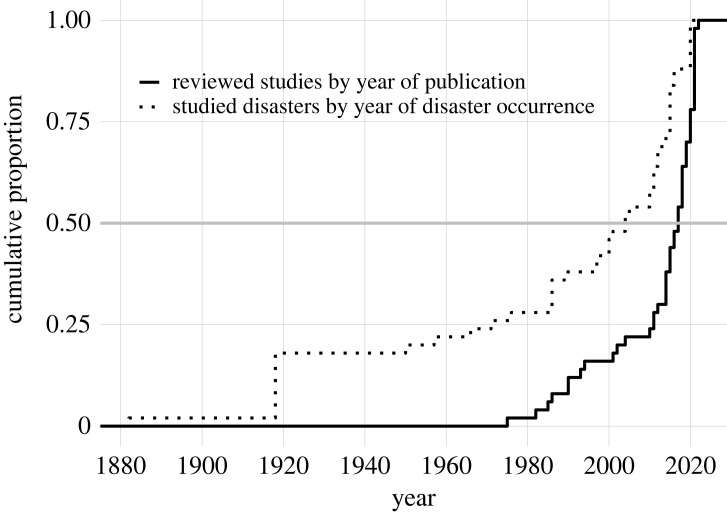
Temporal distributions of the 50 studies reviewed and disasters covered by the studies. The *x*-axis refers to the year of publication (solid line) or disaster occurrence (dashed line), and starts from 1880 because the earliest disaster covered by the reviewed studies was the 1882 measles outbreak in Iceland. The year range of disasters covered by the 50 studies is wider than the year range of publications, reflecting the recent increase in the body of works that quantified fertility changes after disaster. More than 75% of studies were published post 2000.

**Figure 3.**
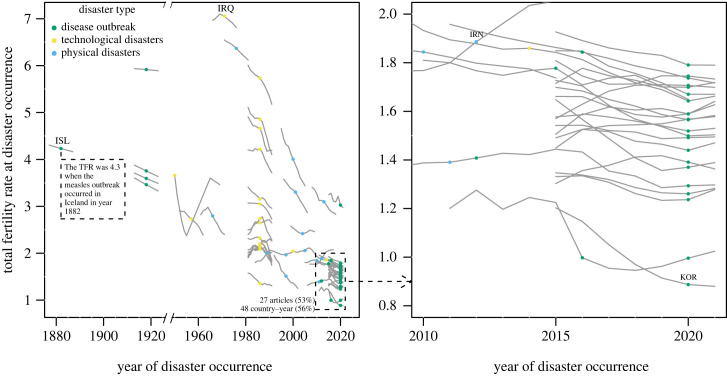
For more recent disasters, the disaster–fertility relationship is studied in a low fertility context. Each marker represents a country matched with a disaster and the country-level total fertility rate (TFR; *y*-axis) at the time of disaster occurrence (*x*-axis). Three markers distinguish disaster types (disease outbreaks, technological disasters, and physical disasters). To show the demographic context of each studied disaster, grey lines represent the time series of TFR five years before and after the disaster. For some disaster–country combinations we found more than one study. These include the 2015 Zika epidemic in Brazil (six papers), the Covid-19 pandemic in Italy (four papers) and the US (two papers), the 2011 Japanese earthquake (three studies), the 1918 influenza epidemic in the US (three papers), and the 2010 Chilean earthquake, 2004 Indonesian Tsunami, and the Chernobyl accident in Ukraine and Belarus (two papers). This information on the number of papers is not reflected in the graph. TFR data before 1950 comes from Statista (interpolation was conducted to fill single years series). TFR data after 1950 comes from the World Population Prospects 2022.

**Figure 4.**
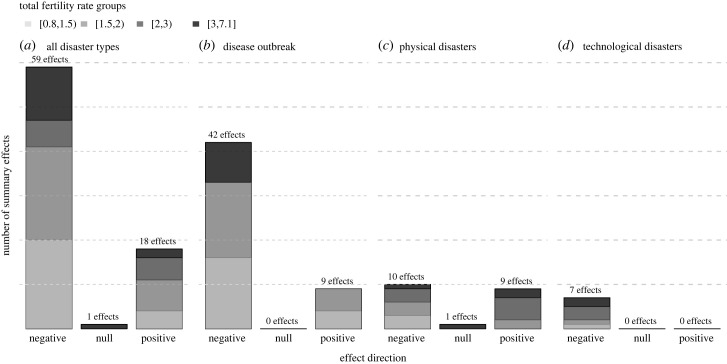
Directions of effect by country-level total fertility rate (TFR), for all disasters (*a*), disease outbreaks (*b*), physical disasters (*c*), and technological disasters (*d*). To assess the potential relationship between the effects' direction and the fertility context, bars are divided according to country-level TFR groups. Due to small sample size and to warrant consistency of cross tabulations, the Chi-squared test ignored rows and columns with no counts. The figure is based on 78 effect sizes available with information on effects direction, regardless of their magnitude and statistical significance. From the 82 summary effect sizes we had, four were not used because they were either associated with multiple countries (i.e. no single TFR could be assigned) [36,37] or they did not have corresponding TFR information for the year of disaster occurrence [38,39].

**Figure 5.**
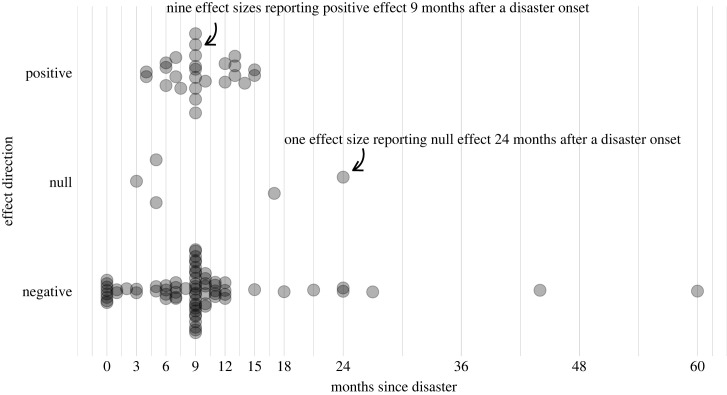
Directions of fertility effects reported at different time lags from disaster onset. Each grey circle represents an effect size available with information on the effect's direction, regardless of the effect's magnitude and statistical significance. Effect sizes are classified into either positive [fertility increase], negative [fertility decline], or no change (*y*-axis), and aligned by time lag of post-disaster fertility follow-up (*x*-axis). Assuming that follow-up began randomly, we can expect that the temporal distribution of positive, negative, or zero effects will give ideas about the temporal dynamics of disaster impact. One model of such dynamics often invoked is ‘initial fertility decline followed by fertility rebound'. We used all 105 effect sizes for which information on time at effect measurement was either reported or could be estimated at monthly intervals.

**Figure 6.**
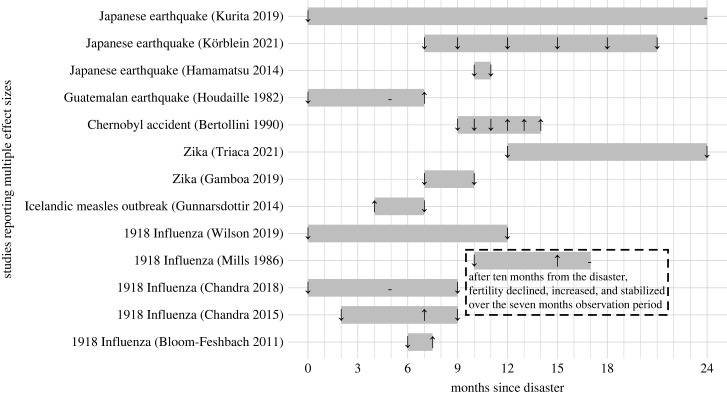
Studies reporting effect sizes across multiple time points since the onset of a disaster. For each unique study (*y*-axis), effect directions (decrease [↓], no change [−], increase [↑]) are aligned by time lag of post-disaster fertility follow-up (*x*-axis). The grey horizontal bars represent the time window of the observation period, which starts from the first post-disaster month at which an effect direction is reported and ends at the last post-disaster month at which an effect direction is reported. We used 41 effect sizes extracted from 13 studies that reported fertility effects across multiple post-disaster time points.

**Figure 7.**
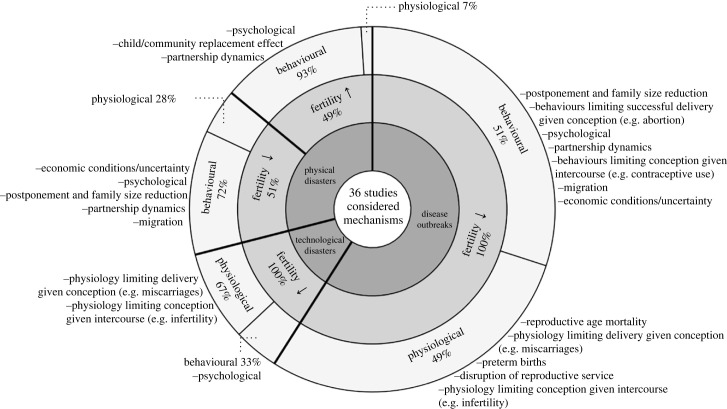
Mechanisms driving the impact of disaster on fertility based on 36 studies that mentioned at least one possible mechanism underlying the disaster–fertility relationship. A total of 123 mechanisms mentioned by the 36 studies were classified by disaster types, effects direction, and whether a mechanism can be considered as behavioural or physiological. Mechanisms most frequently mentioned are shown on the top of each group, and mechanisms mentioned only once are not shown. For a full list of mechanisms by disaster types and fertility effect directions, see electronic supplementary material, tables S1 and S2.

The unit of analysis was either studies (figure 2), countries matched with disasters (figure 3), effect sizes (figures 4–6), or mechanisms suggested by studies (figure 7). While there were 50 studies reviewed, not all studies covered just one country or reported only one effect size or mechanism, leading to different samples depending on the unit of analysis.

### (e) Fertility effect of disaster

In this review, a fertility effect refers to ‘changes in live birth counts after a disaster’. If an effect is reported as a numeric value, then it conveys both information about the direction and the magnitude of effect (i.e. effect size). The direction of an effect can be smaller [negative] or larger [positive] than 0, or not different from the control group [null]. Where possible, we extracted standardized effect sizes (e.g. percentage difference or change). If there were multiple effect sizes, we identified a summary effect, which is either the effect size reported from an author's preferred analysis or the effect size mentioned as the main finding of a study.

From the 50 studies, we extracted 108 data points on fertility effects, of which 82 were summary effects. Out of the 108 effects, only 76 had further information about magnitude because some studies did not report fertility effects in numeric forms for multiple reasons. For example, a multi-country study reported effect values from the study's preferred analysis for only select countries with large and statistically significant effect [51], or, in some studies, fertility of case versus control groups was reported side by side across multiple time points without a single summary effect [40,52–54]. For these studies, we could still extract information on effect direction based on the authors' conclusion and/or visualization of effects. We could not extract any information on effects from one study [55] that only compared *post-disaster* fertility of the affected population with the unaffected population, making it impossible to separate the impact of disaster from any pre-existing difference between the compared populations.

It was not feasible to summarize effect sizes via meta-analysis, due to differences in research designs, effect size types, duration of effect measurement, as well as the unavailability of information necessary for meta-analysis. Instead, we conducted various alternative analyses. First, we used a vote-counting method [56], which compares the numbers of studies with positive versus negative effects using a binomial/sign test. The null hypothesis states the equal chance of positive versus negative effects based on binomial distribution. All available information on the direction of point estimates regardless of statistical significance entered vote-counting, because vote-counting can be misleading if a subjective decision (e.g. an arbitrary alpha level for statistical significance) is used to determine a vote. Second, we used Chi-square tests and log-linear models to examine differences in the counts of positive versus negative effects by disaster types and countries’ total fertility rate (TFR) at the year of disaster. Third, we analysed effect direction by time lag from disaster to the beginning of outcome measurement, first by pooling all effect directions reported in the studies and then by confining to 13 studies that examined fertility effect across multiple post-disaster time points. We calculated the time lag in months. Lastly, we examined patterns in effect sizes among those reported in comparable metrics, specifically % change in fertility adjusted for seasonality and time trends in fertility. There were 18 such effect sizes.

### (f) Mechanisms

Out of the 50 studies, 36 postulated at least one mechanism underlying the fertility effects of disaster.

We classified mechanisms into 13 groups, which were also categorized as either behavioural or physiological mechanisms (electronic supplementary material, tables S1 and S2). Of the 13, the behavioural mechanisms were: child/community replacement effect (no. 1), economic conditions/uncertainty (no. 2), migration (no. 3), psychological (no. 4), partnership dynamics (no. 5) and postponement/reduction of family size (no. 8). The physiological mechanisms were: disruption of reproductive services (no. 9), preterm births (no. 12), and mortality of men or women of reproductive age (no. 13). There were two additional mechanisms that could be behavioural or physiological depending on contexts: conception given intercourse would be affected behaviourally via (no. 6) contraceptive use but also physiologically via (no. 10) infertility, and successful delivery given conception would be affected behaviourally via (no. 7) abortion but also physiologically via (no. 11) miscarriage.

Although it is challenging to integrate the 13 mechanisms into one framework, we follow the approach taken by a previous review on fertility after wars, humanitarian crises and displacement [13]. Here, different pathways by which fertility changes were seen as a combination of indirect (distal) and direct (proximate) determinants of fertility, following the frameworks proposed by Davis & Blake [57] and later by Bongaarts [58]. Among the 13 mechanisms we identified, the proximate mechanisms which can affect fertility directly are (i) intercourse that is driven by partnership dynamics (no. 5), (ii) conception given intercourse (no. 6,10) and (iii) successful delivery given conception (no. 7,11). Partnership dynamics can influence fertility either through marital or coital rate; conception given intercourse can be affected by contraceptive use or women's ability to conceive (i.e. fecundity). Mechanisms affecting fertility through successful delivery given conception encompass different types of pregnancy termination (both related to the decision to terminate - through abortion- and involuntary termination, for example miscarriage). The remaining mechanisms are either indirect ones (no. 1–4, 9) or ones that cannot be easily classified as direct or indirect (no. 8, 12–13). For example, economic conditions are a clear example of an indirect mechanism through which disasters can affect the proximate determinants mentioned above. Conversely, a broad group of mechanisms frequently mentioned in the studies, which we call ‘postponement/family size reduction’, is not easily classifiable, as it could be an outcome of an interplay of both indirect determinants (e.g. economic conditions) and proximate determinants (e.g. partnership dynamics) included in mechanisms groups no. 2–7. Similarly, preterm births and reproductive age mortality (i.e. either women or men of reproductive age) cannot be easily classified according to the proximate determinants framework.

## 3. Results

### (a) What has been studied, and how?

#### (i) Temporal and geographical scope of reviewed studies

The 50 studies were published between 1975 and 2022 and covered 24 different disasters, which occurred between 1882 (the measles outbreak in Iceland) and 2020 (the COVID-19 pandemic) across 52 countries. Our study sample is biased toward studies that examined certain disaster types and regions. Twenty-two out of the 50 studies (44%) were on either the Zika epidemic, the 1918 influenza pandemic, or the COVID-19 pandemic, and the majority of these studies were based on samples from Brazil, Italy, Japan and the US. In this regard, 44% of the research on the disaster–fertility nexus has thus far focused on disease outbreaks from middle- and high-income countries. Indeed, 10 out of the 50 studies covered the US, and more than half (52%) covered either Brazil, Italy, Japan or the US.

The literature is relatively young, as most works were published over the last two decades (solid line, figure 2). More recent disasters were more likely to be studied, with more than half of the studies covering post-2000 disasters (dashed line, figure 2). This trend resulted in shorter time lags between disaster and publication for more recent disasters, such as the COVID-19 pandemic and the Zika epidemic. Such a trend may reflect an increase in both the interest in the topic and data availability, in particular for individual-level and panel data [89].

However, the recent expansion of the literature does not correspond to the actual distribution of recent disasters and disaster risks. For example, our search did not identify studies on several natural disasters that occurred during 2000–2019 with more than 100 000 casualties [2], notably the 2008 Cyclone Nargis in Myanmar, the 2005 Pakistan earthquake and the heatwaves in Europe (2003) and Russia (2010). The deadliest natural disaster of the last two decades, the 2004 Indian Ocean Tsunami, affected 12 countries in Asia and Africa. Still, only two studies in the reviewed literature examined the fertility consequences of this disaster, and only in Indonesia [84,85].

To further understand the demographic context of the literature, we examined the distribution of countries studied according to country-level total fertility rate (TFR) and year of disaster occurrence. Figure 3 displays which countries were covered by the 50 studies, according to the disaster year and the country-level total fertility rate (TFR) at the time of disaster. The lines trace the time series of a country's TFR 5 years before and after the disaster. Figure 3 shows that our study sample covers a wide range of TFR levels (from 0.8 to 7.0) and temporal trends. Fertility exhibits substantial declines before and after the disasters—particularly for those before 2010—making it important to consider time trends of fertility within study designs. This is also important for disasters occurring in low fertility contexts, where fertility continues to fluctuate yearly.

Figure 3 speaks again to the strong concentration of studies in recent years (also figure 2), and additionally shows that the concentration is among low and relatively stable fertility contexts represented by countries in the Global North (figure 3, right panel). Most of these countries are included in multi-country studies. One multi-country study that contributed significantly to this pattern [51] exemplifies a trend that favours multi-country comparative analyses—especially in high-income countries—based on the availability of harmonized and comparable data such as the Human Fertility Database (https://www.humanfertility.org) and the Short-Term Fertility Fluctuations Data Series (www.humanfertility.org/Data/STFF). Studies on more recent disasters (especially post-2010) also concern relatively low fertility contexts with TFR below two (figure 3, right panel), driven by research on the COVID-19 pandemic in high-income economies and the Zika epidemic in Brazil and Colombia. Among the whole 50 studies, there are very few studies conducted in settings with TFRs above four (figure 3, left panel); among them, most studies were on physical or technological disasters that occurred in the Global South.

#### (ii) Methods for measuring fertility effect of disaster

In nearly two-thirds of the studies, one population serves as its own comparison group (within-population comparison; table 1). Fertility was compared (i) between before (control) and after (case) the disaster or (ii) between what is expected under the assumption of no disaster (control) and actually observed (case). In the rest of the studies, at least two populations, one not affected (control) and the other affected (case) by the disaster were compared before and after the disaster (between-population comparison). In most of these studies, the subnational area(s) hit hard by a disaster served as the ‘affected’ group, then compared with other subnational areas or the national-level average.

Studies employed three types of effect sizes to quantify the difference in fertility between case and control. Counts or rates were the most common type. The fertility measure of choice was calculated for each of the case and control groups over a certain period of time, and then compared either in terms of raw differences or ratios. Only some of these studies reported information on size of the population from which the births were counted (i.e. person-years of exposure) for both case and control—the information required for meta-analysis of effect sizes on counts or rates data. Other less common ways to calculate effect sizes included correlating mortality at its peak with fertility 9–10 months after [38,39,61] or calculating differences between average births of case versus control groups [51,59,78,82], either in the aggregate or in rates.

The majority of the reviewed studies (84%) presented information on the onset of a disaster at least in monthly units, a prerequisite for defining the post-disaster period. The onset of a disaster was defined in two ways. First, researchers defined the onset of disaster prior to observing data, by referring to the time point when a disaster is known to have created an acute and major shock. Information on the timing was available *a priori*, if (i) a disaster is unanimously said to have occurred on a specific day or across a few days (e.g. earthquake) or (ii) the researcher makes an explicit assumption about when the disaster likely had the most impact on fertility. The latter was a frequent choice if there was a lag between the emergence of hazards and their development into a disaster, as in the case of the COVID-19 pandemic where the lockdowns are often regarded as its onset of impact in respective countries. Second, some studies analysed monthly data on proxies of exposure to disaster (e.g. disease incidence rate, Internet search for the disaster), to inductively identify the month at which the exposure (or the perceived exposure) peaked [36,41,42,61].

### (b) How does fertility change after disaster?

#### (i) Fertility effects

The effect of disasters on fertility was generally negative, even when controlling for secular time trends. Out of the 108 effect sizes for which information on direction was available, 77 were negative and 26 were positive. According to the binomial test of the assumption that the counts of negative and positive effects are even, the proportion of negative effects was 0.66 (95% confidence interval [CI] = 0.57 to 0.79, *p* = 0.006). Similarly, among the subset of 81 summary effect sizes (effect sizes upon which each study based its main conclusion), the proportion of negative effects was 0.71 (95% CI = 0.59 to 0.82, *p* < 0.001). The tendency for negative effects became weaker when we further restricted to 46 effect sizes that adjusted for time trends, but the majority of effect sizes (59%) were still negative (95% CI = 0.43 to 0.73, *p* = 0.302).

*Effect directions by disaster type and fertility context.* Figure 4 displays the distribution of fertility effects for all disaster types (pooled) and for each disaster type separately. To assess the potential relationship between the effects' direction and the fertility context, bars are divided according to the country-level TFR at the time of disaster. Negative effect sizes dominated the studies on disease outbreaks and technological disasters, but not the studies on physical disasters. Negative and positive effects were reported across all TFR levels at similar proportions (figure 4*a*), and the same was true within disease outbreaks (figure 4*b*) and physical disasters (figure 4*c*). This comparison was not possible within technological disasters, for which no positive effects were reported (figure 4*d*). We further examined the counts of effects by direction (positive versus negative), TFR and disaster type using log-linear models. Among the models with different combinations of variables, the model with the interaction between TFR and disaster type yielded the highest goodness of fit as suggested by lowest model accuracy metrics (electronic supplementary material, table S3). This finding is likely driven by the effect sizes from physical disasters, because the majority (78%) of positive effects reported from physical disasters pertained to contexts with the TFR above 2.0 (figure 4*c*). These results again suggest that the direction of effects may not differ by the TFR of the studied populations.

*Effect directions by time points.* Figure 5 shows the direction of the fertility effects (positive, null and negative) by time since the disaster. Follow-ups began on average 9 months from the disaster onset (interquartile range = 6–10 months). Negative effects were most commonly reported between 0 and 12 months after the disaster, especially at 0 (i.e. at the onset) and 9 months after the disaster. There were less positive or null effects reported. Nonetheless, both negative and positive effects were most commonly reported at 9–10 months from the disaster onset.

Among the 13 studies that reported effect sizes across multiple time points since the onset of a disaster (figure 6), only one study on the 1918 influenza in India reported a pattern of fertility decline, rebound above baseline, and stabilization [43]. In five studies births declined then rebounded; however, exact time points at which negative and positive effects were reported varied across the studies [36,38,40,43,44].

*Magnitude of fertility effects.* There were 18 effect sizes that reported effects as percentage changes and adjusted for seasonality and time trends in fertility (electronic supplementary material, table S4). Most of them were negative, ranging from −1.7 to −43%. Only one effect was positive, showing a 9.5% increase. From these studies, two interesting patterns emerge. First, for studies that examine precisely the same context and disaster, effect sizes and directions agree very closely. One example is the 2011 Japanese earthquake, where two studies found a 9–10% reduction in fertility. Second, for studies that analyse the same disaster and country context but different sub-national regions, effect sizes for smaller areas tend to be larger in absolute terms. For example, four studies examine the effect of Zika in Brazil: a study on the Northeast region alone found a 25% decline in births, whereas a broader analysis of the 36 largest cities found only a 7.7% decline, and two yet broader analysis (one of all municipalities with at least one case, and another at the national level) found a very similar 1.7% and 1.9% decline. This is possibly due to publication bias towards studies which find large effects—regional analyses with null effects are less likely to be published, leaving only those with large effects. Similar patterns are found among the studies on the 1918 influenza pandemic in the US and COVID-19 in Italy.

### (c) Mechanisms

Among the 50 reviewed studies, 36 (72%) referred to at least one possible mechanism to explain the observed effects of disasters on fertility. Figure 7 shows how frequently studies invoked (if they did any) behavioural or physiological mechanisms to explain findings. For example, for disease outbreaks, studies considered behavioural and physiological mechanisms at a similar frequency, whereas physiological mechanisms were considered more often for technological disasters. It should be noted that, although studies on the two disaster types are shown to concern with mechanisms underlying negative effects, this does not mean that these studies did not consider any mechanisms regarding positive effects if they mentioned any. Rather, this means that the studies concluded an *overall* negative effect based on their analyses.

Among the 20 studies on disease outbreaks that mentioned at least one mechanism and reported a negative impact on fertility, over half considered behavioural mechanisms. These included contraceptive use, postponement of childbearing, reductions in completed family size, or abortion, as well as psychological factors (e.g. fear of health risks). Some of the studies also considered physiological mechanisms, such as those preventing successful delivery (e.g. miscarriage), reproductive age mortality and preterm births. Most of these 20 studies on disease outbreaks were on the Zika epidemic and the 1918 influenza pandemic, but notably, the former was mostly concerned with behavioural mechanisms (87% of the time) and the latter with physiological mechanisms (80% of the time). This diverging pattern reflects how different characteristics of risks associated with each disaster can result in the consideration of different mechanisms by researchers.

This pattern also manifests itself in technological and physical disasters. For these disaster types, the mechanisms considered in each study were dominated by either behavioural or physiological mechanisms, but not both (figure 7). Researchers interpreted fertility change from technological disasters mainly through the lens of physiological mechanisms relating to conception and successful delivery, perhaps because all the disasters studied involved the spread of hazardous chemical substances. By contrast, physical disasters were postulated to drive fertility change mainly through behavioural mechanisms. Here, mechanisms involving positive fertility effects have been most explicitly considered, including psychological factors (e.g. attachment), child/community replacement effects, and partnership dynamics (see electronic supplementary material, table S1 for specific mechanism(s) each study mentioned).

## 4. Discussion

This systematic literature review demonstrates that disasters reduce fertility, albeit heterogeneously by disaster type. Specifically, while physical disasters resulted in either positive or negative fertility responses, for disease outbreaks and technological disasters the summary effects were uniformly negative. When positive effects were found for physical disasters, they were more likely to occur in higher fertility contexts (TFR > 2.0). However, we find this tendency was not strong enough to support the oft-postulated notion that positive fertility effects are in general more likely in higher TFR countries [6,7]. In addition, we found little evidence that the direction of fertility effects differs by time since disaster: negative effects were reported anywhere from the onset to five years after the disaster, suggesting that positive effects are likely to be short-term, if any, even for physical disasters [32].

The concentration of positive effects from physical disasters may reflect differences in how risks develop and are perceived during these events. Physical disasters are characterized by a relatively acute, recognizable shock. As such, one can postulate that the waning of risks would also be perceived relatively easily, leading to a higher probability of fertility increase during the recovery period. By contrast, disease outbreaks and technological disasters bring risks that are often unclear, prolonged or difficult to track (e.g. the spread of viruses, toxic materials such as radiation, etc.). Such uncertainty, together with the direct and often long-term physiological effects of virus infection or toxic materials on fecundity and health in general, may underlie the predominant pathways by which disease outbreaks and technological disasters are thought to reduce fertility.

Our findings are corroborated by studies that were not included in the present review because they did not meet our eligibility criteria [32–34]. A recent study [90] published six months after our literature search also concluded fertility reduction during 5 years after various disasters in African countries. In this study, the evidence for fertility decline was clearest for disasters with prolonged periods of uncertainty, a finding that is in line with our above interpretation. Fertility declined more in areas already vulnerable to the risk of droughts, suggesting that continuing uncertainty from disasters could be a contributing factor to the observed fertility decline in response to disasters. On the other hand, the average change in fertility was either unclear or non-substantial after short-duration disasters, such as floods, earthquakes, tropical cyclones, other storms and epidemics. Of note, the definition of epidemics in that study only included *rapid* outbreaks, and excluded endemic diseases such as HIV/AIDS. In other words, the study operationalized epidemics conceptually similar to physical disasters in terms of the time scale of risk development. It can thus be summarized that disasters involving higher uncertainty about risks may contribute to fertility reduction, whereas the evidence of fertility decline is weaker for relatively short-duration disasters. As such, the study suggests that the general findings from our review may hold even in relatively high fertility contexts such as in Africa.

While we find clear uniformity in the direction of summary effects for some disaster types, overall the results were heterogeneous. Among physical disasters, the direction of effects was variable, but also even within the same disaster, and even within the same population groups. As an example of between-population heterogeneity, one study on the 2004 Indonesian tsunami conducted in the most severely affected regions found a strong negative effect [84], while another cross-regional study found a fertility increase [85]. Some studies also examine within-population heterogeneities in the fertility effects of disasters, as summarized in table 1. This heterogeneity urges more future research to consider contextual factors driving the divergence of fertility effects, especially given that disaster vulnerability is highly unequal both across and within countries and socioeconomic groups [91].

We also note that the current literature is biased toward research on certain contexts and disasters, in spite of a substantial expansion of research on the disaster–fertility nexus during this period. Particularly noticeable were gaps for low-income countries, and for certain high-casualty disasters which occurred during the last two decades. This is consistent with a preoccupation with disasters causing high economic losses rather than high casualties [92]. Similarly, our analysis of the magnitude of fertility effects suggests a potential publication bias for studies finding large impacts, since sub-national effects were on average larger than national ones. We could not formally test publication bias due to the heterogeneity in disaster types, timeframes, methods, and types of effects reported. However, it is unlikely that negative effects were published at a higher rate than positive effects, since both positive and negative effects were hypothesized *a priori*.

In the remainder of this paper, we identify common limitations from the reviewed literature and summarize them into six practical recommendations for future research. While not prescriptive, these recommendations can help researchers produce and evaluate evidence on causal effects—essential for a field involving humans where experimental evidence is problematic. Whenever possible, we highlight examples of methods from reviewed studies that can be usefully applied for future research.

First, data infrastructures for collecting data in low-resource settings should be supported, particularly during disasters (Recommendation 1). Conducting regular yet well-spaced population censuses is essential for monitoring the effect of disaster on fertility and mortality; however, doing so remains difficult for many low-income countries [93]. Researchers can take an active role in improving data collection by openly appraising the quality of data and data infrastructure used in their research. Doing so would also help assess the risk of bias in their findings. For example, errors surrounding the under-counting of births due to delayed reporting, house births in case of historical data, and outmigration due to disaster were frequently reported in the reviewed studies. Another common source of bias was the omission of pre-disaster fertility data from women who died from the disaster, particularly in cases where pre-disaster fertility was determined by retrospective self-reports from survivors [84]. This case underscores a need for continued and regular monitoring of demographic data. Other possible efforts to overcome the bias present in the current literature include mapping data availability (e.g. demographic and health surveys, and multiple-indicator surveys) and disaster occurrence jointly. This initial mapping would identify existing gaps that can be addressed readily by future studies.

Improved data availability, in turn, will lay the groundwork for more evidence building on how disaster affects fertility in a global perspective. Findings that do not meet the conventional threshold of ‘statistical significance’ should be published (Recommendation 2), as long as the data, analytic approach, and estimated effects are clear (accompanied by the necessary information for meta-analysis and standardization, e.g. person-year exposure for birth counts [65]). Doing so will not only facilitate open science practices in general, but also give a much-needed opportunity to compare the fertility effects of disasters across different contexts.

Future research should present more detailed information on the population characteristics of comparison groups, or at least indicate if such information is not available (Recommendation 3), and wherever possible, analytically adjust for differences between compared groups that may confound the disaster–fertility relationship (Recommendation 4). Researchers, either implicitly or explicitly, assume that groups that are being compared are generally similar aside from the exposure to disaster, otherwise known as the ‘homogeneity assumption’. Satisfying this assumption helps rule out alternative explanations that attribute observed fertility changes to factors unrelated to disaster. When comparing fertility within populations before and after a disaster, researchers should recognize that pre-disaster fertility differences across subpopulations may contribute to post-disaster gaps. Likewise, researchers should acknowledge that the effects of disasters on fertility may be direct and indirect. The frequency of sexual intercourse in a population may be lowered in the aftermath of a disaster due to physical damages or poor health (direct effect). At the same time, birth rates may decline due to changes in population structure (indirect effect), for example, if individuals of reproductive age were more likely to die due to the disaster than the overall population. Less than 10 out of the 50 studies tested specific threats to population homogeneity before and after the disaster, or even presented background characteristics of each compared group. These threats included both spatial (spillover of fertility effect to control cohorts [45]) and temporal (conflation with concomitant events such as economic crises, which was indirectly tested in one study by examining socioeconomic gradients in fertility effect [65]). A few studies used synthetic control methods to create a comparable control [46,76,94].

Disasters do not happen in a vacuum, and fertility within countries is rarely stable. For example, fertility can rapidly fall in countries undergoing the demographic transition, and oscillate in countries that have already achieved low fertility. Many of the studies reviewed here did consider and account for these context-specific fertility trends, and such efforts would continue to benefit the literature in future (Recommendation 5). One common technique is to analytically adjust for seasonal fertility patterns and long-term fertility time trends. In our reviewed studies, the majority (80%) addressed seasonality, mostly by comparing fertility at least one year apart matched in months or by including month dummies in regression analyses. However, only half (52%) reported effect sizes that took time trends into account. This is problematic, given that a still large portion of the studied disasters occurred in contexts of declining fertility.

Lastly, one of the most basic requirements for causal inference in this area is to establish that the disaster actually preceded the observed fertility change [95]. However, defining the onset of a disaster can be difficult. For example, for disease outbreaks, the timing of the first case may be very different to the timing of the maximum effect on the population. In eight out of the 50 reviewed studies, there was no clear indication of the timing of disaster onset, the timing at which fertility measurement began, or both. As an alternative, some studies identified the disaster onset *post hoc*, by subtracting 9–10 months from the period associated with the largest or statistically significant fertility effect. This approach might be useful as an exploratory technique, but is subjective and vulnerable to questionable research practices such as *post hoc* hypothesizing.

Instead, some authors made explicit their beliefs about the timing and magnitude of fertility impacts, a practice that could aid in evaluating the evidence at hand and building theory (Recommendation 6). Some studies stated how large an effect is expected to be, in the form of either qualitative statements (‘little to no effect’ [65]) or even setting a threshold of what the researchers considered to be a large enough effect size given the fertility trends of the study population [71]. Regarding timing, one study on the 2011 Japanese earthquake [47] defined a ‘disaster impact period' based on authors’ reasoning about when the disaster's impact is likely to manifest, and another study on the 1997 Red River flood [80] specified ‘pre-disaster’ and ‘post-disaster’ periods for precise months and years based on known information about the progression of the disaster. In doing so researchers clarify not only the expected timing of shock, but also after how long the impact of a disaster is expected to last. The latter is particularly important since different mechanisms will be operative across different timeframes. For example, if a specific disaster would significantly increase the mortality of pregnant women, one might hypothesize an immediate reduction in births. Very long lags between exposure and fertility impacts could indicate a longer-run change in fertility intentions via postponement effects. Unfortunately, very long time lags also increase the likelihood that other post-disaster events will confound the estimation, making it difficult to attribute observed fertility differences to the disaster of interest. In nearly half of the reviewed studies, outcome measurement began after a lag *longer* than 9–10 months, the average length of gestation. However, among such studies, only one provided a rationale for why a longer lag was chosen [85], demonstrating the need for more explicit hypotheses surrounding the timing of fertility outcome measurement.

## 5. Conclusion

Across the 50 studies examining the changes in live birth counts after disasters, we found that disasters generally have a negative fertility impact, depending on disaster type. If any, studies showing an increase in fertility were more likely to come from studies on physical disasters, which included avalanche, drought, earthquake and tsunami, flood, hurricane and typhoon in the reviewed studies. We also identified 13 distinct mechanism groups which researchers have considered as underlying the fertility effects of disaster. By contrast to the common belief that disasters are more likely to increase fertility in contexts with already high fertility, we found little evidence to suggest that the underlying fertility level was an important predictor of the direction, timing or size of fertility impacts. While this may be because no relationship exists, it may also be due to biases in the literature towards studying high-income countries or high-cost disasters. This bias may also arise from a lack of standardization by methods and timeframes in this literature, leading to a small number of studies across which to compare. We thus need more studies from high fertility contexts, and also more efforts to measure fertility effects comparably and across multiple time points. Doing so will also help disentangle key mechanisms of fertility effects which are numerous and complex. To encourage moves toward a more robust literature on this critical topic, and eventually toward evidence-informed theory and policy, we offer six recommendations for future research: (1) increase efforts to build and maintain data infrastructure for the collection of quality data in resource-limited settings; (2) nurture a publication culture that incentivizes reporting of results (including null results) to be compared across contexts; (3) collect and report characteristics of compared groups; (4) analytically address threats to the homogeneity assumption; (5) adjust for seasonality and time trends in fertility; (6) be explicit about when and how the expected fertility effects of disasters are likely to occur. Given the predicted increase in disasters—particularly in the context of global climate change—even subtle changes in fertility in response to disasters may have far-reaching consequences in population structure. Thus, the importance of studying how disasters affect fertility is higher than ever, and should remain a major priority in the research community across disciplines, from demography to population studies, economics, anthropology, sociology, public health and epidemiology.

## Data Availability

The original data extracted from the 50 studies, and detailed information about the screening and selection procedure are available at the project website (https://osf.io/dpgqf/) [96]. Supplementary material is available online [97].
